# A multiple case study of intersectoral public health networks: experiences and benefits of using research

**DOI:** 10.1186/s12961-016-0082-7

**Published:** 2016-02-11

**Authors:** Anita Kothari, Charmaine McPherson, Dana Gore, Benita Cohen, Marjorie MacDonald, Shannon L. Sibbald

**Affiliations:** School of Health Studies, The University of Western Ontario, Labatt Health Sciences Building, Room 222, London, ON N6A 5B9 Canada; School of Nursing, St. Francis Xavier University, Antigonish, Box 5000, NS B2G 2W5 Canada; College of Nursing, University of Manitoba, Winnipeg, MB R3T 2N2 Canada; School of Nursing, University of Victoria, Victoria, BC V8P 5C2 Canada; Department of Family Medicine, The University of Western Ontario, London, ON N6A 5B9 Canada

**Keywords:** Decision-making, Health equity, Health promotion, Intersectoral, Knowledge, Networks, Public health, Public health systems, Research, Third sector organisations

## Abstract

**Background:**

Network partnerships between public health and third sector organisations are being used to address the complexities of population level social determinants of health and health equity. An understanding of how these networks use research and knowledge is crucial to effective network design and outcome evaluation. There is, however, a gap in the literature regarding how public health networks use research and knowledge. The purpose of this paper is to report on the qualitative findings from a larger study that explored (1) the experiences of public health networks with using research and knowledge, and (2) the perceived benefits of using research and knowledge.

**Methods:**

A multiple case study approach framed this study. Focus group data were collected from participants through a purposive sample of four public health networks. Data were analyzed using Framework Analysis and Nvivo™ software supported data management. Each network had the opportunity to participate in data interpretation.

**Results:**

All networks used published research studies and other types of knowledge to accomplish their work, although in each network research and knowledge played different but complementary roles. Neither research nor other types of knowledge were privileged, and an approach that blended varied knowledge types was typically used. Network experiences with research and knowledge produced individual and collective benefits. A novel finding was that research and knowledge were both important in shaping network function.

**Conclusions:**

This study shifts the focus in the current literature from public health departments to the community setting where public health collaborates with a broader spectrum of actors to ameliorate health inequities. Both formal research and informal knowledge were found to be important for collaborative public health networks. Examining the benefits of research and knowledge use within public health networks may help us to better understand the relationships among process (the collaborative use of research and knowledge), structure (networks) and outcomes (benefits).

## Background

The public health (PH) sector, mandated with protecting the community’s health through surveillance and population health assessment, disease and injury prevention, and health promotion and protection efforts, requires diverse partners such as government, the private sector, academia, and primary care. A key area of partnerships and collaborations is with the third sector, which can include organisations such as, but not limited to, non-governmental organisations, local associations, community groups, and charities. The third sector is uniquely positioned to address the social determinants of health for a variety of reasons. Many third sector organisations engage with the social determinants of health directly, sometimes called ‘social care’. For example, organisations support people struggling with the impacts of poverty, food insecurity, or gender-based violence [1, 2]. They often work with the most marginalized and stigmatized in society and, through this, have a targeted impact on reducing health inequities [2, 3]. Third sector organisations can also be very connected to the communities that they serve, which is useful in helping PH initiatives reach communities and build community collaborations [2, 3]. Furthermore, third sector organisations frequently engage in direct healthcare delivery (e.g. mental health services, care for the elderly) and health promotion (e.g. harm reduction for sexually transmitted infections and substance use, support for parents and young children). A scoping review of third sector activities in Scotland found that these organisations were vital in promoting a healthy population through active engagement in health policy, healthcare provision, and health promotion in the areas of children and parental wellbeing, substance use and sport [4].

While there are many forms that PH and third sector collaborations can take, an emerging and increasingly prevalent configuration is that of the PH network, which is formed between PH and third sector organisations [5–8]. Networks can be defined generally as “*an arrangement of individuals and/or organizations that are linked through connections that range from informal relationships to formally agreed protocols*” ([9], p.1). A review of related literature suggests that collaboration between people or organisations to achieve a shared objective that no partner can achieve alone, and the cooperation of autonomous organisations to provide programmes or address a common population health issue is at the heart of the definition and functioning of a network [10–12]. Some other noted characteristics of networks are that they work to achieve their collective vision and mission rather than only the priorities of their individual organisations, members cooperate as equals rather than operating in a hierarchical framework, and governance is less top-down and more based on mutual trust [13]. It has been suggested that intersectoral collaboration between PH and third sector organisations is particularly amenable to a network approach because both types of organisations address large, complex issues that cannot be tackled alone. Networks break silos, encourage lateral relationships, and have the flexibility to generate creative collaborations to address these issues [13].

In order for these emerging collaborations to achieve a significant impact in PH and reduce health inequities, it is critical to understand how these partnerships and networks use knowledge. As the evidence-based practice movement has demonstrated, sound research is a cornerstone to developing and implementing effective programmes [14–16]. However, to date, little attention has been given to how PH and third sector collaborations, and PH networks in particular, use research and knowledge [14–16].

Research on the use of research in third sector organisations is only just emerging, but has already documented a number of barriers. A systematic scoping review of research and knowledge use in this sector in the United Kingdom revealed that, while third sector organisations were interested in using research to inform their initiatives, many were not able to implement this fully and, as a result, research was often used to satisfy funder requirements rather than contribute to programme development and improvement [17]. Some organisations were constrained by limitations in time, staff expertise, material resources, ability to apply the research to their particular context, and an organisational culture that placed more value on experience and/or tacit knowledge than research knowledge [17]. There are also well known barriers to using research in the PH sector, particularly among PH practitioners. A systematic review by Orton et al. [18] in 2011 of research use by over 1,300 local, regional, and national PH decision-makers in countries with universal healthcare coverage found that the extent to which they used research to inform PH decisions was not clear. Research was only seen as one source of knowledge and influence among other factors such as financial sustainability, strategic fit and pressure from stakeholders. The impact of research was indirect and competed against many other factors [18]. Even when research is used, it is not always used in ways that are effective – there is some evidence for PH decision-makers using research to justify decisions that were already made, rather than informing the decision-making process [18, 19].

Given these trends in research and knowledge use among both the PH and the third sector, it is important to understand whether PH networks are effective structures for sharing research and different kinds of informal knowledge, since effective use of knowledge can contribute to favourable PH outcomes [18]. As noted by others [20], community-based networks need to grapple with how to combine and evaluate diverse types of knowledge, or they may need to reconcile research-based guidance that is at odds with local needs. The goal of this study was to determine the extent to which networks are effective structures for research and knowledge sharing and utilization. In terms of operational definitions for this work, research was considered to be findings resulting from the systematic investigation of a phenomenon, using scientific principles, and knowledge was seen as information and ideas. The purpose of this paper is to report (1) the experiences of PH networks with using research and knowledge, and (2) the perceived benefits of using research and knowledge.

## Methods

### Study design

In this research, we used a multiple case study approach. Case study methodology supports the investigation of socially complex phenomena and its surrounding context, thereby providing a rich, holistic understanding of phenomena [21]. Accordingly, each PH network was treated as an individual case. Both qualitative and quantitative data were collected; the qualitative data were gathered using focus groups and the quantitative data were collected with social networking questionnaires designed to understand connections between people. In this paper, we focus primarily on the qualitative data related to research use; the social networking data will be reported on in a separate paper. Ethics approval was obtained from Research Ethics Boards at The University of Western Ontario and St. Frances Xavier University, and from study sites’ ethics review committees, where required. Standard procedures to ensure confidential and ethical protection of the participants and the study data were used, such as obtaining signed informed consent.

### Sample

Networks were eligible to participate if they (1) addressed a population health or PH issue, (2) included at least two member organisations from the third sector, and (3) had members who were over 18 and spoke English. Networks that were diverse in terms of geographic area, membership size and level (local, regional, provincial) were selected purposefully. Networks were recruited through PH units, based on established relationships between research team members (AK, MM and CM) and the directors of PH units or equivalent in three Canadian provinces across the country for geographical diversity. Initial contact involved an e-mail to the director or equivalent of each organisational unit. Upon confirmation of interest, the director identified a network associated with the unit, and network members were invited to attend a preliminary information meeting with a team member to discuss project goals and assess interest in participation.

### Data collection

Data were collected from four networks (hereafter called Sites 1–4) between February and July 2011. At each site, network members attended a focus group and completed a series of questionnaires related to their connections with others in the network. The focus group moderator (DG, AK or CM) used open-ended questions to ask members about their experiences related to working in a network, how new knowledge came into the collaboration, and how this knowledge influenced decisions, including interacting with other individuals in the broader community. The Chair of the network had the opportunity to review and comment on the focus group guide in advance. As a result, the guide was adapted to the local network context and language based on the Chair’s recommendations [22]. Focus groups were used to achieve a network-level response to the issues and lasted about 90 minutes.

### Analysis

Focus group data were audio-recorded, transcribed, de-identified and systematically analyzed by a subgroup of research team members (DG, SS and BH) using framework analysis [23, 24]. Framework analysis uses a conceptual scaffolding approach that facilitates the development of inductive and deductive themes from both participant data and the research questions [25]. After reading all the transcripts, each subgroup member independently created preliminary codes based on a common transcript, identifying core themes based on questions asked in the focus groups (their experiences related to working in a network, how new knowledge came into the collaboration, and how this knowledge influenced decisions, including interacting with other individuals in the broader community). Coders were also open to inductive themes emerging from the transcript. Then, the subgroup came together to compare the preliminary codes and collapse them into an initial thematic framework. The subgroup went through a second round of this process to identify sub-themes, create definitions, and map examples of data onto these themes. Once coding of the thematic framework was finalized (Table 1), one subgroup member recoded all transcripts using the final codes and definitions. In other words, the team member systematically went through each focus group transcript to assign data segments from the transcripts to the relevant code in the thematic coding framework (Table 1). Another subgroup member checked the coded data for consistency. All research team members subsequently came together in a face-to-face meeting to interpret the coded themes; consensus on interpretation was achieved through discussion. The Nvivo 9™ software, specific to qualitative data, was used to manage the data. Team members presented findings to each network and network members had the opportunity to comment on and discuss the interpretation of the data. Through this member-checking strategy our interpretations are established as reasonable and credible by study participants.

**Table 1:** Public health networks focus group coding framework

Code	Subcode	Response examples
1.0 Functions of Network	1.1 Internal (functions to members)	1.1.1 Resource sharing
		1.1.1.1 Knowledge
		1.1.1.2 Strategies
		1.1.1.3. Skills
		1.1.1.4. Experience
		1.1.2 Ensuring member accountability to topic
		1.1.3 Continuing professional development
		1.1.4 Support + motivation
	1.2 External (function in broader community)	1.2.1 Advocacy (in/direct)
		1.2.2 Public outreach/intervention
		1.2.3 Public education
2.0 Information Entry	2.1 Who (who brings info in)	2.1.1 Individual members
	2.2 What	2.1.2 Expert consultant
		2.2.1. Research literature
		2.2.2. Experiential knowledge
		2.2.3 Anecdotal evidence (community-based)
		2.2.4. Network derived empirical data (survey, evaluation tool)
		2.2.5. External empirical data (e.g. local stats)
	2.3 How (tools/processes used)	2.3.1 Ad hoc (spontaneous conversation)
		2.3.2 Electronic mailing
		2.3.3. Personal emails
		2.3.4. Phone
		2.3.5. Face-to-face meetings
		2.4.6. Workshop/conferences
		2.4.7 Online community interface
		2.4.8 Literature review
		2.4.9 Designated information distribution within network
	2.4 Tools/processes desired/needed	2.4.1 Government connection
		2.4.2 Webinars
		2.4.3. Sharepoint
		2.4.4 Diagnostic support
		2.4.5. A1C tool
		2.4.6 Academic connection to bring in research capacity
		2.4.7 Full time staff to synthesize research
3.0 Information Influence on Network Function	3.1 Influence on individual network members	3.1.1 Clarify roles
	3.2 Influence on network function	3.1.2. Influences members’ actions in their parent orgs
		3.1.3 Helped identify skills
		3.2.1 Increase collaboration between members
		3.2.2 Focus topic interest
4.0 Conflict/Disagreement Within Network	4.1 Nature of disagreement/conflict	4.2.1 Use of consensus
	4.2 Process by which it is resolved	4.2.2 Withdrawal of members
	4.3 Impact on network	4.2.3 Respectful discussion
		4.3.1 Improves network focus on issues
		4.3.2 Improve network problem-solving processes
		4.3.3 Provides a good debate
5.0 Barriers to Network Function	5.1 Political	5.1.1 Network restrained by conservative learning of member organisation
	5.2 Time	5.1.2 General conservative political climate
	5.3 Communication	5.2.1 Constraints on members’ time to look at new info
	5.4 Information management	5.2.2 Constraints on members’ time to participate in network
	5.5 Lack of common goals for network	5.3.1 Poor communication system between network members
	5.6 Other	5.4.1 Challenge of using existing tech (e.g. Sharepoint, Google docs)
		5.4.2 Lack of an individual to help synthesize evidence
		5.4.3 Difficulty not duplicating resources
		5.5.1 Lack of consensus about overarching goal of network
		5.5.2 Philosophical differences between members
		5.5.2 Lack of consensus about role of network
		5.6.1 Different jurisdictions represented (geography of network)
6.0 Facilitators to Network Function	6.1 Inter personal facilitators	6.1.1 Synergy among partners
	6.2 Network-level facilitators	6.1.2 Frequent contact between members on other projects
	6.3 External facilitators	6.1.3 Trust/respect between members
		6.1.4 Common goals of members for network
		6.2.1 Interdisciplinary nature of network
		6.2.2 Network builds on existing relationships of member orgs
		6.3.1 Context/location of network itself (e.g. big city: more info + funding potential)
		6.3.2 Trust of communities that networks work with
7.0 Influence of Network on Members as Professionals	7.1 Knowledge	7.1.1 Personal (e.g. helps members broaden their perspectives, focus on ‘who they are’ in public health)
	7.2 ‘Networked’ network	7.1.2 Professional (e.g. members get help, advice, feedback on ideas + new info about public health issues)
	7.3 Camaraderie	7.1.3 Community/context (e.g. deepens members understanding of issues in community)
		7.2.1 Good experience for newer members to learn from others
		7.2.2 Network good for making new contacts
		7.2.3 Increased connection to community through network contacts
		7.3.1 Reduces isolation in working on a difficult issue
		7.3.2 Motivates members in their own work
8.0 Network Context	8.1 Origins	Unique narratives for each network
	8.2 Development	
	8.3 Everyday network activities	

## Results

Overall, the response rate for focus group participation was 47% (29/62). In part, this moderate response can be traced to the largest network, Site 1, a provincial network where many participants use teleconference to join network meetings and therefore were not able to participate in the on-site focus group. The response rates by network were Site 1: 9/28 (32%); Site 2: 3/5 (60%); Site 3: 10/16 (63%); and Site 4: 7/13 (54%). The characteristics of each network are described in Tables 2, 3, 4 and 5, and summarized in Table 6.

**Table 2:** Site 1 characteristics

Site 1 was a provincial-level network that focused on population health, i.e. the social determinants of health and health inequities. The group acted as an informal think-tank, bringing together high level officials from various PH-related organisations (health, research and allied sectors) in the province to have strategic discussions around the inequities agenda, and exchange knowledge and information about what was happening in member organisations around this issue. This network began in 2006 in response to the recognition that there needed to be an explicit place to discuss population-based approaches to health. Because this group was self-directed, volunteer-based and existed as an entity apart from any particular organisation, it had a large amount of autonomy in determining its direction and priorities.	
As of December 2010, its membership consisted of 28 members from various PH-related organisations in the province, including representation from government, planning bodies, alliances, universities and non-governmental agencies. Membership was somewhat fluid, as various people from other sectors were periodically invited into the network.

**Table 3:** Site 2 characteristics

Site 2 was a small network that was action-focused on public outreach and education of vulnerable groups around a specific chronic condition. The network conducted frequent education sessions and screening for disease risk at English as a Second Language and French schools, reaching a large proportion of the immigrant population in Ontario city. The network was created in 2009 by three members who met at an annual conference and realized there was a large gap in condition-specific prevention services in their geographic area. The network was volunteer-based, i.e. not mandated by any particular organisation. From 2009 to the focus group conducted in June of 2011, the network was still in its pilot stage for its education and screening activities, and was periodically reflecting on and refining their processes.	
The five members in this group represented three organisations: a regional PH organisation, a local community education programme and a national chronic conditions organisation. One member was an epidemiologist in charge of data management, while the other four members were in charge of coordination, execution and follow-up from events. Network members remained accountable to their member organisations and had to justify their involvement and time invested in this network’s activities.

**Table 4:** Site 3 characteristics

Site 3 was a medium-sized voluntary network of 16 members whose objective was to improve communication, coordination and collaboration among organisations working toward enhancing active living in the region. The network was created as a result of a meeting between the PH department, the provincial department of health and community partners in response to low physical activity rates in the geographical area. The network has been active since 2009, and as of 2011 had representation from government at the local, regional and provincial levels as well as school boards, PH organisations and non-profit associations.	
As of 2011, the network had created a strategic plan and two task groups, and had carried out multiple evaluations of its functioning and progress. It was in the process of carrying out public outreach and advocacy activities. For example, in 2011, the network focused on creating an active living/recreation database and website, and advocating for an active transportation plan with local government. The network also acted as a knowledge resource and networking source for members. New organisations were occasionally invited to join when the network felt that the organisation was a good fit with the network’s purpose and objectives. Decisions about membership were made by consensus within the network.

**Table 5:** Site 4 characteristics

Site 4 was a regional-level, community-based, not-for-profit network organisation with 13 members. The network was founded in 2007. Its key purpose was to promote, support and advance sustainable development in the region. The network used a four-pillar approach (cultural, economic, environmental and social) to sustainability, considering each initiative in terms of these impacts. Part of the network’s mission was to support the area in becoming a model sustainable community through the engagement of its citizens. It worked with the community (e.g. through community engagement forums, brainstorming sessions) to determine priorities for sustainability initiatives, and then conducted research and advocacy on these topics.	
Key activities of the network were carried out through Action Teams that were issue-specific coalitions around topics such as food security, poverty and the natural environment. The network also conducted educational activities and consultations in the areas of research, advocacy, economic innovation and leadership. The voluntary membership base was diverse, including representatives from civil society, businesses, cultural groups, PH and the broader health sector, and environmental organisations.

**Table 6:** Network characteristics

	Site 1	Site 2	Site 3	Site 4
Purpose	To share information, resources and work on activities that further population health and reduce inequities	To create awareness of an individual’s risk of developing a specific chronic condition and to provide follow-up to those individuals	To improve communication, coordination and collaboration among partners working toward improving and enhancing active living	To promote, support and advance sustainable development
Function	Knowledge exchange and indirect advocacy	Service delivery through community outreach	Knowledge exchange, leadership and advocacy	Leadership and advocacy, partnership formation
Geography	Rural + Urban	Urban	Rural	Rural
Structure	Informal	Formal	Formal	Formal
Type	Provincial	Municipal	Regional	Regional
Age	6 years	3 years	2 years	6 years

**Table 7:** Summary of findings

Thematic findings	Site 1	Site 2	Site 3	Site 4
Experiences of PH networks using research & knowledge:				
A range of knowledge sources used (explicit and tacit)	+	+	+	+
Knowledge used in a holistic, not hierarchical, fashion	+	+	+	+
Research and knowledge actively introduced/gathered by network members	+			+
Benefits of research & knowledge for PH networks:				
Knowledge exchange transformed network functioning	+			
Research & knowledge supported network activities		+		
Research & knowledge focused the role of network			+	
Research & knowledge central to purpose and function of network	+			+
Research & knowledge from network helps members in their professional roles	+		+	+
Research & knowledge from network helps members personally	+		+	+
Research & knowledge from network helps members understand community			+	
Working together on research brings trust and knowledge of others’ skills	+		+	

Findings are organised under the two main research purposes of understanding the (1) experiences of PH networks with using research and knowledge, and (2) perceived benefits of using research and knowledge. In each of these sections, we draw the reader into our interpretation of themes using guiding questions and illustrative quotes from participants.

### Experiences of PH networks with using research and knowledge

#### How is research and knowledge obtained and used?

Although the four PH networks had very diverse structures and functions, all of them used a variety of forms of research and knowledge, ranging from formal scientific research articles to anecdotal community knowledge. The PH networks did not attempt to hierarchize research and knowledge; rather they were used in a holistic, blended way to meet the knowledge needs of the networks.

In Site 1, research and knowledge came primarily from members themselves. Members would bring in topics of discussion for the network, and occasionally bring in a guest presenter on particular topics as well. The topics were largely contextual; they related to how members’ parent organisations were dealing with inequities, or what was happening in other networks of which the members were also a part. This type of political and organisational knowledge was used for members to have strategic discussions about how inequities are dealt with in provincial PH organisations, and in what direction they should be headed. Members stressed that they did not consider themselves a research network. More formalized knowledge was occasionally circulated by members, for example, tools, websites, reports or research articles. However, this was done primarily for the sake of interest rather than to support primary discussions.

“*For the most part, people have their own mechanisms for maintaining their knowledge base on population health research, so we haven’t really tried to do that here. It’s more of a strategic discussion around what people know*.” (Site 1)

At Site 2, a network primarily focused on implementation of particular activities, a wide range of research and knowledge was used to help inform network practice. For example, when deciding on a risk assessment tool, the network used a literature search to help identify existing tools. Members then used their experiential knowledge to evaluate how feasible the tool would be to implement with their target population.

“*And getting back to the* [network name] *model, we looked at some research of other similar kinds of models … so we had to decide: what was really feasible for the population that we were trying to reach and what was feasible in terms of the time we were going to put into it? …*” (Site 2)

During public outreach activities, Site 2 used a combination of different types of knowledge to learn how to provide the best outreach possible. This knowledge came from community consultations to learn about barriers to healthy living, experiential knowledge about particular needs of the communities they served, and anecdotal knowledge in the form of spontaneous feedback from these communities about the outreach activities. The network also gathers community data directly using a survey administered to participants during outreach activities to track chronic disease risk and follow-up. Throughout the network’s activities, members consulted with guidelines and toolkits on best practices from their own organisations or external organisations (e.g. Health Canada), relevant research studies, and external consultations with PH experts when necessary.

At Site 3, members also used a blend of different types of research and knowledge to inform their work around issues relevant to the network, such as active transportation. The network used local knowledge in this case to learn about active transportation in their area. For example, they conducted surveys with municipal councils and local government on their awareness of active transportation. They also incorporated several questions that were relevant to active transportation into the Canadian Community Health Survey that was being done by the regional health authority. In addition to their own data gathering, the network also used local statistics to learn about towns in the region and their level of participation in and use of active transportation. Members attended a 2-day active transportation summit to learn about current research and best practices. That being said, all the research data used were grounded in network members’ knowledge from their daily work. The research data did not necessarily take a forefront position in network discussions:

“*I guess we’re working on a lot of assumptions based on the evidence that we see in our daily work. I don’t know how much we’re like, “oh you got to see this evidence, I have this research.” We have our physical activity pieces of research that, I mean we’re not always talking about it, but we read it and we’re very familiar with it, and the understanding of our health stuff. We know this stuff. We kind of work not only through* [network name] *on those but kind of through all of our daily activities, they’re always research based…no, we’re not always bringing up research here at the table.*” (Site 3)

At Site 4, network members actively sought out research and knowledge on sustainability initiatives that could potentially be used in their own community. For example, when thinking about creating bike lanes in their own city, members researched benefits and impacts of bike lane construction in small and large cities in Canada and Europe. They acknowledged that research may not always be tailored to the particular context of the community, and so network members used the approach of finding the best available knowledge most comparable to their community. The network accessed community data directly through brainstorming with community members in community engagement forums. The network also used other types of knowledge to help form their conceptual perspectives of sustainability and to engage with the community on this issue:

“*And I think our discussions around multi levels, like the conceptual: what is the sustainability? what is a sustainable lifestyle? what would that look like in* [city name]*? It’s also I think what are the different pragmatic options? So we’re relying on some scientific expertise you know, is it windmills? Is it geothermal? …so we’re researching that and we’re also researching models of social change. So I think we spend a lot of time talking about how does change occur and then we also do a social analysis of the community, like where can we push to make change? Where are there blockages to change? So I think it’s conceptual levels. It’s at different sort of information and scientific knowledge levels. It’s also that sort of relational circles of influence knowledge*.” (Site 4)

### Benefits of research and knowledge for PH networks

#### How does research and knowledge use affect network functioning?

For all sites, the way research and knowledge were used in the networks interacted with and affected the way they functioned. In Site 1, knowledge exchange was absolutely key to the network because it was its primary function. When members talked about the network’s purpose, they described it as a place for idea sharing and critical discussion about health inequities. Members could then use these ideas and perspectives to inform work in their own organisations. Because inequities were often not part of the organisational priorities of individual organisations, members viewed this group as a unique and much needed space to meet with ‘kindred spirits’ and have high-level discussions about the inequities agenda. While originally the network may have been more focused on specific actions, knowledge exchange about inequities became so critical that it transformed the objectives and function of the network:

“*I think that we probably had at the very beginning some thoughts about being a network that undertook to do things, but I think more and more, it is the exchange of knowledge and the ideas and people … so it’s a network that brings together people from a whole bunch of different organizations who then can go out and do things. The network itself probably isn’t structured or funded or resourced, or whatever to actually do … but certainly it brings together people to talk and then the other organizations can carry out or work on the areas…*” (Site 1)

Members stressed that the central work of the network was not really held by the network itself; rather, it was in how individual members could take away research and ideas to their own organisations and continue pushing the inequities agenda from there. From this perspective, the knowledge exchange that was taking place certainly helped advance the mission of the network:

“*…often times what I find is that the level of discussion and information that is shared at the meeting actually allows people to tailor it then to whatever their mandate or how they can actually advance the agenda from their sector’s perspective*.” (Site 1)

At Site 2, research and knowledge use were not the primary purposes of the network; however, it was fundamental to the initiation and continuation of the network’s prevention activities. Research and other types of knowledge played a key role in the selection of the risk assessment tool and decisions about how to implement public outreach activities. The ultimate decision of which risk assessment tool to use also helped to lend credibility to the network because the tool had a strong research base:

“*…I think the CanRisk tool was a real good find for* [network name] *in terms of okay, yea, this is best practice, it’s statistically validated in Europe over a ten year study, and it’s gained the attention of the Public Health Agency of Canada, and they’re doing a pilot study in 13 sites across Canada, and it’s the best tool…*” (Site 2)

The statistical data on participant demographics and risk levels collected during outreach activities also helped to tailor the ongoing activities of the network. At the time of this study, aggregate data analysis was taking place to help the network determine its impact and plan for the future.

Members in Site 3 reported that activities around acquiring knowledge or doing research together tightened the role of the network, forced them to formalize it more, and also helped to assess particular strengths or skill sets of members that could be used in an effective way. For example, a knowledge-gathering exercise that was done around management of the web portal helped members determine how much the network would be responsible for and in what way.

“*I thought that exercise too around trying to acquire more information on active transportation was a good assessment of who’s got capacity, in terms of human resources, to dedicate time to do additional research, and even fiscal resources to acquire that knowledge … we identified who else is out there trying to collect information, or already has the data, … let’s make sure we don’t duplicate*.” (Site 3)

Site 4 data revealed some similarities to Site 1 in that research and knowledge was central to the purpose and function of the network, although this network’s mandate was around sustainability.

“*To some extent it’s the nature of the organization in that it looks for information and it’s a relatively new topic, sustainability …it’s an inherently complex system so you cannot look at one piece on its own, in its own definitive way. So if the need to gather information disappeared then you could frankly retire from being chair and we can close up the board and do something else*.” (Site 4)

Like Site 1, Site 4 looked outwards to gather research and information about initiatives that could be adapted to their own community or organisations. Site 4 was committed to implementing strategies and advocating for change in the community as well as researching them. Research was seen as a key piece of Site 4’s mandate, but it could also only go so far; the network also came together for specific activities to make their community a more sustainable place:

“*Next Thursday night we’re all getting together in special suits and white paint buckets and we’re going to paint the bike lines because all of our research didn’t go anywhere with it*.” (Site 4)

#### How does research and knowledge use benefit individual members?

Site 1 participants reported that the feedback they received at meetings (in the form of helpful advice, reactions, and suggestions for their own work) is extremely helpful for them as professionals and also helps motivate them to continue their work.

“*This group’s been critical to me in terms of very intelligent knowledgeable people sitting around the table, giving feedback and suggestions on all of those things, so whenever something like that comes up, bringing it to the* [network name] *is the first thing I think of, in terms of getting a lot of useful and helpful advice and reactions to it, so that’s been the biggest role of the* [network name]. *In terms of my work, it’s a group that I can bring this stuff to and get really knowledgeable feedback*.” (Site 1)

Another member described how, through the process of working together, people developed an awareness of each other’s skill sets and areas of expertise. Finally, someone at Site 3 mentioned that, since subgroups worked together on research, a deeper level of trust among members was developed.

Members at Sites 1, 3 and 4 found that the network benefited them definitively in terms of the amount of knowledge they gained from the network. This knowledge could have multiple facets: personal, professional, and community-based. In the case of personal knowledge, some responses were that it helped define ‘who they are’ in PH, opened their minds, and that it prompted them to make changes in their personal lives.

“*Well, I don’t know, the only thing I could say is that I think in a way it helps define who you are as a professional because there isn’t any professional discipline of population health inequities champion…*” (Site 1)

There was significant data related to how the knowledge gained at the network was useful for members professionally. Often, the knowledge exchange, advice and research presented at the network was valuable for members and allowed them to tailor it and use it for their own professional purposes. Some members were exclusively participating in the network for the professional knowledge benefit or to see where their own organisation and perspective fit. Other members had already taken the knowledge gained at the network and applied it in their careers, e.g. with other organisations with whom they worked.

“*What I was thinking about is that there aren’t a lot of forums where you actually bring together a variety of different sectors and a variety of different content experts that really allow us to address some of the issues of public health that require that real collaborative effort … I find that the level of discussion and information that is shared at the meeting actually allows people to tailor it then to whatever their mandate or how they can actually advance the agenda from their sector’s perspective*.” (Site 1)

“*I got my own business, and the knowledge gained here is applicable in an economic sense. We’re fostering relationships. We’re just keeping an eye on cutting edge technologies that I might want to go and involve my own business with. It’s very helpful*.” (Site 4)

Finally, a member of Site 3 mentioned that participating in the network increased his/her community-based knowledge: knowledge of where the community is at with a particular issue and what needs to be done to bring about change.

“*…I’m in community development. That’s kind of a professional practice and so for me what’s really exciting is to see the sharing of the knowledge, the thinking about this as a collaborative effort. How can we share our resources and do a better job of moving this forward, which doesn’t mean we all keep doing what we’re doing at home, but how can we keep moving this forward so it helps to strengthen from my perspective the communities that we live in…?*” (Site 3)

## Discussion

Collaborations between PH departments and third sector organisations have been put forward as a way to address the complex issue of health inequities. There is a gap in the literature, however, about how research and knowledge are used in these collaborative network structures. Four PH networks were examined to understand their experiences in using research and knowledge, and the benefits perceived therein (Table 7).

The first important finding from this study was that all networks used published research studies and other types of knowledge to accomplish their work. Research and information played different but complementary roles: in some cases research was used to provide context or select interventions, while in others, knowledge was used to understand research findings. Sources of knowledge ranged from locally-derived evaluations and data, to guest speakers and colleagues. The analysis also identified varied types of knowledge, including organisational, anecdotal, political, experiential, content-related and knowledge about how different content areas influence each other. A recent scoping review to understand the types of knowledge and evidence that PH decision-makers use aligns with these findings. Developing a typology of tacit and explicit knowledge, Boyko [26], in 2004, further categorized explicit knowledge as expert-based, locally-derived or formalized. Tacit knowledge was found to be either experiential or emotional [26]. Other studies support the finding that tacit or informal knowledge is an important input for identifying programmatic solutions for PH issues [27, 28]. Longstanding discussions about the multiplicity of knowledge types and their application are also prevalent in other fields (e.g. education [29] and psychology [30]). What is most important to understand through future studies, however, is when PH networks use different knowledge types, for which types of decisions and why.

The analysis also pointed out that neither research nor other types of knowledge were privileged over the other. This finding is incongruent with the general trend in the health sector to assign primacy to systematic reviews derived from randomized controlled trials based on the hierarchies of evidence paradigm. However, in their extensive ethnographic study of primary care clinicians, Gabbay and le May [31] found that practitioners and teams developed ‘mindlines’ to guide practice rather than using research. These mindlines were constructed as a composite of research, experience, tacit knowledge and information from opinion leaders and others. These knowledge types were holistically negotiated and individually embodied but collectively reinforced through interactions with peers. Our study hints at a similar process given the blended approach of varied knowledge types. Further, the network infrastructure supports social interactions that are characteristic of mindlines; the network facilitates on-going contributions, discussions, group reflections and learnings such that a shared understanding of the network topic is built. In a recent systematic review of the mindlines concept the authors put forth that *“…mindlines can be accurate and useful in a local setting, and provide useful predictions, despite not being construed according to … the EBM* [evidence-based medicine] *paradigm*” ([32], p.8). The authors also raise the point, however, that controlling knowledge creation through the nurturing of a network may not even be possible. At the very least, we suggest that researchers and practitioners might shift their terminology from ‘evidence-based practice’ to ‘evidence-informed practice’ or ‘knowledge-based practice’ to more accurately reflect PH activity.

Another important finding from this study was that research and knowledge were important in shaping the function of the networks. The idea that knowledge creation, acquisition and sharing might have a prominent role to play in how a network carries out its work is not well understood in the larger networks literature. Research has mostly been focused on understanding how network structures or relationships between network members achieve knowledge outcomes such as generating or adopting knowledge [33]. Our study extends this view, and suggests that future studies ought to focus on understanding how knowledge flow, or how the properties of knowledge, have an impact on network function. With this understanding those who can provide research and knowledge, such as university partners or PH departments, can support optimal network performance. This finding also has implications for the field of knowledge translation where current strategies being implemented in the practice setting do not consider the influence of research use on unit function.

The last important finding from this study speaks directly to PH practice: network experiences with research and knowledge produced individual and collective benefits. These benefits might be seen as evidence that, as Popay et al. discuss, PH networks are “*…opening up new spaces within local systems for public health work…*” ([34], p. 339). When speaking about population health and equity issues in particular, collaborative cross-sectoral work is imperative for progress, and networks might be one example of how to do this. The PH collaborative literature tends to centre on community development with the characteristic of keeping groups and community members continuously engaged. The network approach, however, is more flexible in that it allows for an ebb and flow of membership, as needed. Our study also demonstrated some integration of PH activity with research, further supporting the argument that PH organisations might consider additional investment in these inter-organisational structures to support the sharing of the diverse knowledge required to tackle the challenge of health inequities.

Findings from this work need to be considered in light of study limitations. The low response rates at Sites 1 and 4 suggest that the findings from those two networks may not reflect a true aggregate response, however a diversity of views were expressed given the different network profiles. We acknowledge that those who did participate might be more likely to express positive experiences related to their network. Further, the study design does not permit uncovering insights about experiences with research and knowledge over time; only a cross-sectional snapshot of network activity is reported here. Strengths of this work included using a pragmatic analytical technique – framework analysis – that supported team analysis, the participation of diverse networks across the country to capture a range of perspectives, and the inclusion of a process to include network members in a discussion about findings, adding credibility to the interpretations.

## Conclusion

This study adds to the growing body of work in the area of PH and the use of research for decision-making. This study demonstrated that both formal research (peer-reviewed or locally-based) and informal knowledge are important in collaborative and cross-sectoral networks. Our study setting expands the usual approach of focusing on PH departments, and moves the discussion to the community where PH works closely with other third sector organisations. By shifting this focus we hope to encourage other researchers to also consider the broader spectrum of actors who work with knowledge to do PH work and improve the population’s health.

This study also adds to the networks and communities of practice literatures. The analysis demonstrated that research and knowledge influenced the function of the networks. We also demonstrated that benefits, both at the network and individual level, were identified in participant discussions about research and knowledge. This line of inquiry is exciting, and opens up areas of investigation to further understand the relationship among process (the collaborative use of research and knowledge), structure (networks) and outcomes (benefits).
